# Elinvar-Like Effect Induced by High Lattice Distortion in Zr_6_Ta_2_O_17_ Ceramics

**DOI:** 10.34133/research.0436

**Published:** 2024-08-05

**Authors:** Xiaopeng Hu, Qing Liu, Sai Liu, Yu Zou, Jinwei Guo, Junyao Wu, Wang Zhu, Zengsheng Ma

**Affiliations:** ^1^ Key Laboratory of Key Film Materials & Application for Equipment (Hunan province), School of Materials Science and Engineering, Xiangtan University, Xiangtan, Hunan 411105, China.; ^2^ Key Laboratory of Low Dimensional Materials and Application Technology of Ministry of Education, School of Materials Science and Engineering, Xiangtan University, Xiangtan, Hunan 411105, China.

## Abstract

Super strength and toughness, excellent deformation resistance, and high-temperature service performance are the key factors to determine the practical application of new thermal barrier coatings (TBCs). The limited mobility of dislocations and the internal inherent defects in ceramics will inevitably lead to the decline of strength–plasticity and the reduction of service performance. Introducing preexisting twin boundaries and stacking faults (SFs) or preparing ceramic materials with high configuration entropy has demonstrated to be an effective strategy for enhancing the mechanical properties of ceramics. However, due to the positive thermal expansion coefficient of most ceramics and the remarkable increase of structural disorder at elevated temperature, the problem of elastic softening has become a bottleneck restricting the high-temperature service life of new TBCs. In this paper, the deformation behavior of high configuration entropy Zr_6_Ta_2_O_17_ ceramics at 25 to 1,200 °C was in situ monitored via digital image correlation technique and three-point bending test platform in high-temperature environment. A remarkable Elinvar-like effect appears in the Zr_6_Ta_2_O_17_ ceramic. More interestingly, mechanical deformation dominates the severe lattice distortion (deformation twins, SFs) and the disorder–order transition of chemical order at the atomic scale, while temperature can further enhance the degree of lattice distortion and ordering of Zr_6_Ta_2_O_17_ ceramics. Furthermore, the atomic fluctuations at high temperature promotes the comprehensive improvement of mechanical properties in the Zr_6_Ta_2_O_17_ ceramics.

## Introduction

Seeking an excellent balance of stiffness, strength, and toughness has become a research hotspot of structural materials [1,2]. Unfortunately, high strength and high toughness are usually mutually exclusive [3]. Ceramic materials have natural rigidity [4,5] and strength [6,7], but the poor toughness has significantly restricted its applications [8,9]. At present, as an essential aspect of material design, lattice distortion plays a crucial role in impeding crack propagation and expansion, as well as enhancing the toughness of ceramic materials. This is achieved through local strengthening, alteration of plastic deformation mechanisms, or the pinning effect of dislocations [10–17]. Significant progress has been achieved in the direct introduction of local distortion during the preparation process. For example, preexisting high-density stacking faults (SFs) are introduced in ceramics via flash sintering process, which triggers the activation of the inelastic deformation mechanism of ferroelastic domain switching in advance [18–21]. Excitingly, mechanical deformation and temperature field can also induce lattice distortion to improve the mechanical properties of the material [22,23]. Nevertheless, the most critical thing is that the mechanical properties of most ceramics are negatively correlated with temperature [24,25], especially that the ability to resist the deformation at high temperature is significantly reduced, which is mainly attributed to the positive thermal expansion coefficient and the increase in structural disorder [26,27]. This has become a fatal flaw in ensuring the safe application of ceramic materials in high-temperature environments. At present, some progress has been conducted in the elastic stability of Elinvar alloys. The Elinvar effect is generally attributed to spontaneous volume magnetostriction or magnetic exchange energy [28,29]. In addition, the local perturbation of the Peierls potential caused by strong lattice distortion and atomic-scale chemical ordering can increase the friction of the lattice on dislocation motion so that the material has excellent elastic strain limit, very low energy dissipation, and Elinvar effect [30]. The abnormal elastic modulus mechanism also exists in the ferroelastic phase transition or strain glass transition of some metal ferroelastic, some ferroelectric ceramics, and martensitic systems [31–35]. However, the phase transformation process is accompanied by obvious volume changes, which may not be suitable for high-temperature structural ceramics, especially thermal barrier coatings (TBCs), such as the widely used 7 to 8 wt % yttria-stabilized zirconia (8YSZ) TBCs [36–39].

The development of new TBCs [40–45] is currently hindered by some critical properties issues, including poor mechanical properties and the limited service life, especially the serious decline in mechanical properties at high temperature [46]. It is interesting to note that the new A_6_B_2_O_17_ (A = Zr; B = Ta) TBCs ensure a prolonged service life in high temperature, which is 3 times than that of 8YSZ TBCs [47]. The A_6_B_2_O_17_ (A = Zr, Hf; B = Ta, Nb) oxides with large configurational entropy [48,49] exhibit the unique superstructure, an exceptionally high phase transition temperature (2,250 °C) [50], and outstanding thermal-mechanical properties (*K*_IC_ = 3.36 to 3.78 MPa·m^1/2^, *H* = 12.8 to 15.1 GPa) [51,52]. Previous work [53,54] have confirmed that the A_6_B_2_O_17_ material will be one of the most promising candidate for TBC applications. However, the evolution of mechanical properties of Zr_6_Ta_2_O_17_ ceramics with temperature has never been reported in the literature. More insights into the correlation between microstructure and macroscopic mechanical properties of Zr_6_Ta_2_O_17_ ceramics are strongly required to further understand the evolution mechanism.

In this paper, the mechanical properties of A_6_B_2_O_17_ oxide were predicted by first-principles calculations, and the high-temperature elastic modulus and fracture strength of Zr_6_Ta_2_O_17_ ceramics were evaluated at 25°C to 1,200°C by real-time monitoring of surface displacement and strain evolution during three-point bending (TPB) tests, utilizing a universal testing machine and the digital image correlation (DIC) method. The deformation behavior of Zr_6_Ta_2_O_17_ ceramics is a surprising abnormal temperature-dependent transition, with an Elinvar-like effect, which is confirmed to be related to the microstructure evolution. This work provides the insights into the evolution mechanism of the correlation between microstructure and macroscopic mechanical properties of Zr_6_Ta_2_O_17_ ceramics.

## Results and Discussion

### First-principles calculations and material microstructure characterization

**Fig. 1.**
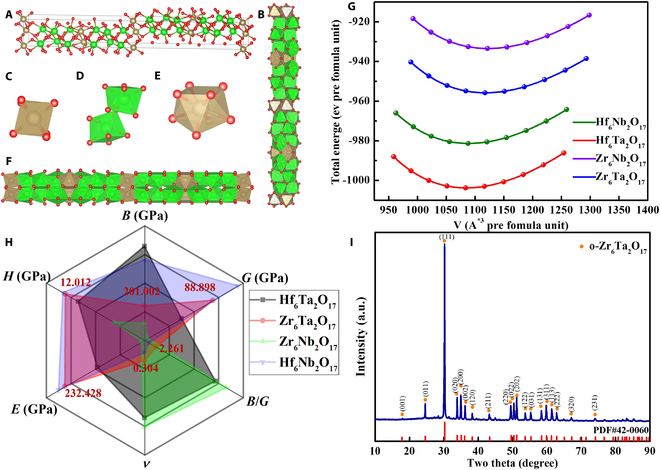
(A) Ball-and-stick models of the A_6_B_2_O_17_ disordered structure (front view). (B) View along the [99] direction. (C) Six-coordinated distorted octahedra. (D) Seven-coordinated distorted capped trigonal prisms. (E) Eight-coordinated distorted bicapped trigonal prisms. (F) View along the [001] direction. (G) Energy-versus-volume curves of A_6_B_2_O_17_ compounds. (H) Performance radar chart of A_6_B_2_O_17_ compounds (the red value is Zr_6_Ta_2_O_17_ compound parameters). (I) XRD patterns of Zr_6_Ta_2_O_17_ ceramics.

The crystal structure of A_6_B_2_O_17_ (A = Zr, Hf; B = Nb, Ta) schematically illustrated in Fig. 1A is an orthorhombic structure with the space group of Ima2 (No.46) at ambient condition. The 6-coordinated distorted octahedra (Fig. 1C), 7-coordinated distorted capped trigonal prisms (Fig. 1D), and 8-coordinated distorted bicapped trigonal prisms (Fig. 1E) form blocks of equidistant cations that are arranged along the *c* axis by corner and edge sharing [48]. The geometrical optimization of the crystal structure was based on the Birch–Murnaghan of equation state. The relaxation of unit cells in A_6_B_2_O_17_ compounds was acquired using Vienna Ab-initio Simulation Package (VASP) software [55]. The energy-versus-volume curves of A_6_B_2_O_17_ compounds are plotted in Fig. 1G, and the lattice constants after equilibrium are also shown in Table 1. The elastic stiffness coefficients of A_6_B_2_O_17_ system, calculated using the strain energy method [56], satisfy the mechanical stability criterion, as presented in Table 2. The bulk modulus (*B*), shear modulus (*G*) of crystal lattice, and the elastic constants of the polycrystalline crystal were calculated by Voigt–Reuss–Hill theory [57,58]. According to the empirical equations (Eqs. 1 to 4), the elastic modulus (*E*), Poisson’s ratio (*ν*), hardness (*H*_v_), and Pughs ratio (*B*/*G*) [59,60] were obtained (see Table 3). The overall performance in Fig. 1F indicates that all materials in the system meet the toughness conditions. Particularly, Zr_6_Ta_2_O_17_ exhibits excellent comprehensive mechanical properties. Furthermore, previous work has also confirmed that Zr_6_Ta_2_O_17_ material has excellent high-temperature service performance [47], so the temperature dependence on the mechanical performance of Zr_6_Ta_2_O_17_ material has attracted much attention. Here, the detectable phase in the ceramics prepared by solid reaction method in this paper exhibits an orthorhombic Zr_6_Ta_2_O_17_ phase without impurity phase precipitation (Fig. 1I) and all elements are homogeneously distributed without composition segregation (Fig. S1).

$$E=\left(9\mathrm{BG}\right)/\left(3B+G\right)$$ (1)

$$\nu =\left(3B-2G\right)/\left(6B+2G\right)$$ (2)

$$H_{V}=E\gamma_{0}\chi_{\left(\nu \right)}$$ (3)

$$\chi_{\left(\nu \right)}=1-8.5\nu +19.5\nu^{2}/1-7.5\nu +12.2\nu^{2}+19.6\nu^{3}$$ (4)

**Table 1.** The crystal lattice parameters of A_6_B_2_O_17_ after optimization

Compounds	*a* (Å)	*b* (Å)	*c* (Å)	*V* (Å^3^)	*α* = *β* = *γ*
Hf_6_Ta_2_O_17_	41.1784	4.9570	5.3124	1,084.3739	90
Zr_6_Ta_2_O_17_	41.5929	5.0070	5.3686	1,118.0575	
Hf_6_Nb_2_O_17_	41.1813	4.9676	5.3218	1,088.6818	
Zr_6_Nb_2_O_17_	41.5645	5.0195	5.3798	1,122.3980	

**Table 2.** The calculated elastic stiffness coefficient (*C*_ij_) of A_6_B_2_O_17_

Compounds	*C*_11_	*C*_22_	*C*_33_	*C*_44_	*C*_55_	*C*_66_	*C*_12_	*C*_13_	*C*_23_
Hf_6_Ta_2_O_17_	344.93	384.39	373.11	62.22	128.78	36.78	137.69	106.93	160.30
Zr_6_Ta_2_O_17_	318.02	352.73	349.68	60.62	115.34	75.23	134.13	106.77	158.00
Hf_6_Nb_2_O_17_	342.99	377.20	360.13	52.08	120.25	95.45	139.73	109.56	154.98
Zr_6_Nb_2_O_17_	318.23	345.88	333.56	50.34	107.43	46.02	136.38	107.50	151.42

**Table 3.** The calculated elastic modulus *E* (GPa), hardness *H*_v_ (GPa), Poisson’s ratio *ν*, and Pugh’s ratio *B*/*G* of A_6_B_2_O_17_

Compounds	B	G	E	*H*_v_	ν	B/G
Hf_6_Ta_2_O_17_	211.420	82.552	219.136	11.688	0.327	2.561
Zr_6_Ta_2_O_17_	201.002	88.898	232.428	12.012	0.307	2.261
Hf_6_Nb_2_O_17_	209.049	94.022	234.950	12.077	0.304	2.223
Zr_6_Nb_2_O_17_	197.948	75.635	201.271	10.780	0.331	2.617

where *B* is the bulk modulus, *G* is the shear modulus, *E* is the elastic modulus, and *v* is the Poisson’s ratio. *H*_v_ is the Vickers hardness, *γ*_0_ is a constant of 0.096, and *χ*_(*v*)_ is a dimensionless function of Poisson’s ratio [61].

### Stiffness temperature dependence with Elinvar-like effect

**Fig. 2.**
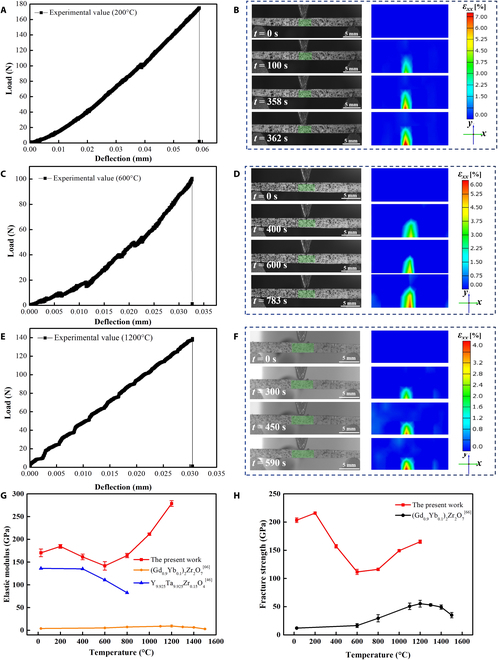
In situ characterization of elastic modulus measurements of Zr_6_Ta_2_O_17_ ceramics during TPB tests. (A, C, and E) Load–deflection curves acquired by the TPB tests at 200, 600, and 1,200°C, respectively. (B, D, and F) Camera photos and contour plots of strain *ε*_xx_ during the TPB tests at 200, 600, and 1,200°C, respectively. The color scale represents change in strain. (G) Evolution of the elastic modulus at different temperature. (H) Evolution of the fracture strength at different temperature.

The TPB experimental procedure of Zr_6_Ta_2_O_17_ ceramics at 200, 600, and 1,200°C was in situ monitored by using a charge-coupled device (CCD) camera with a blue filter. The evolution of the contour plots of strain (*ε*_xx_) of Zr_6_Ta_2_O_17_ ceramics with time is presented in Fig. 2B, D, and F. The Zr_6_Ta_2_O_17_ ceramic can withstand ~7% strain (*ε*_xx_) at 200°C without cracking (Fig. 2B). As the strain (*ε*_xx_) exceeds ~7%, the crack starts to nucleate and propagates along the axial direction. At a higher temperature of 600°C, the strain (*ε*_xx_) of crack nucleation and propagation in the ceramic remains relatively stable, with a similar value of ~6% (Fig. 2D). Nevertheless, crack nucleation initiates at a prominent small strain (~4%) when the temperature rises to 1,200°C, which is 33% lower than that of the specimen tested at 600°C and propagates along the axial direction. Figure 2A, C, and E displays the load–deflection curves obtained by combining the tensile machine and the DIC system at 200, 600, and 1,200°C. Load–deflection curves at 25, 400, 800, and 1,000°C are provided in Fig. S2. There are no plastic deformation during the entire loading process and no obvious jagged shape [21] before fracture, and the load drops sharply to zero at the moment of fracture [62,63] in these load–deflection curves. Additionally, the deflection at 1,200°C is significantly reduced under the same load compared with that at 200 and 600°C. By analyzing the slope of the tangent to the initial linear part of load–deflection curves [64] and the critical fracture load, the corresponding elastic modulus and fracture strength at different temperatures can be obtained (Fig. 2G and H). The specific elastic modulus and fracture strength values at different temperatures are shown in Table S1. As the temperature increases from room temperature to 200°C, the elastic modulus increases slightly from 170 to 184 GPa due to the further densification of the grains. When the temperature rises to 600°C, the elastic modulus decreases slightly to 141 GPa. As the temperature further elevates to 1,200°C, Zr_6_Ta_2_O_17_ ceramics present an exceptionally high elastic modulus (278 GPa). The variation of fracture strength of Zr_6_Ta_2_O_17_ ceramics with temperature is similar to that of elastic modulus. The fracture strength increases from 203 GPa (25°C) to 215 GPa (200°C) and then decreases to 111 GPa (600°C). As the temperature continues to rise, the fracture strength is positively correlated with the temperature and gradually increases to 170 GPa (1,200°C). Compared with TBCs materials in the literatures [46,65] (see Fig. 2G and H), the evolution of elastic modulus and fracture strength of Zr_6_Ta_2_O_17_ ceramics with temperature shows a clear abnormal evolution trend, which is similar to the Elinvar effect [30]. The Elinvar effect is usually attributed to magnetostrictive or magnetoelastic effect [28,29] or second-order phase transition such as ferroelastic phase transition and strain glass transition [33,34], whereas it has not been proved yet that Zr_6_Ta_2_O_17_ ceramic has a magnetic effect and strain glass transformation. Furthermore, stress function renders the reorientation of the ferroelastic domains occurring in ferroelastic ceramics, so lattice tetragonality (*c*/*a* ratio) is generally around 1 [66,67]. Obviously, Zr_6_Ta_2_O_17_ ceramics does not meet this lattice tetragonality condition. Therefore, the Elinvar-like effect in Zr_6_Ta_2_O_17_ ceramics is not caused by either magnetic origin or a ferroelastic phase transition.

**Fig. 3.**
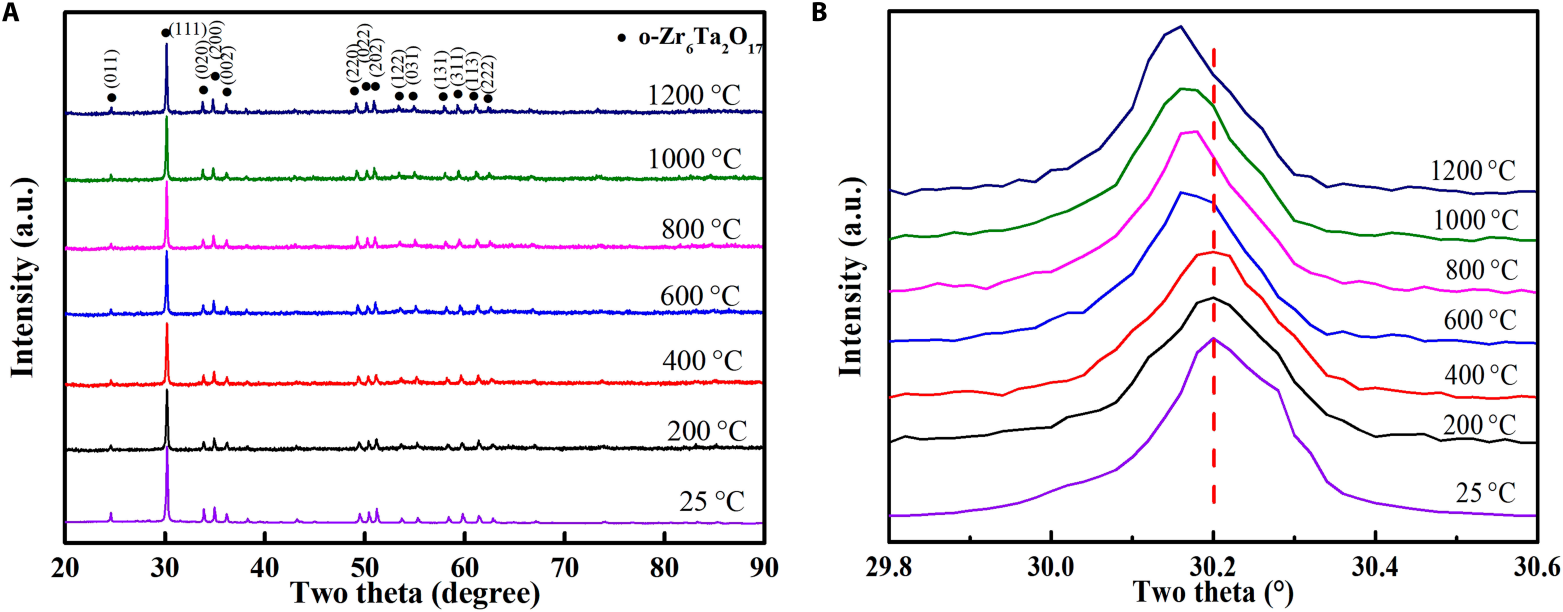
The phase structure and fracture morphology of Zr_6_Ta_2_O_17_ ceramics at different temperatures. (A) In situ HT-XRD patterns of Zr_6_Ta_2_O_17_ ceramics at 25 to 1,200°C. (B) Zoom-in view of characteristic peak (111) around the diffraction angle of 30°.

In situ high-temperature x-ray diffraction (HT-XRD) detection of Zr_6_Ta_2_O_17_ ceramics (Fig. 3A) at 25 to 1,200°C and room-temperature XRD pattern of Zr_6_Ta_2_O_17_ ceramics after TPB tests at different temperatures (Fig. S3) further reveal that the characteristic peak is a typical structure of orthorhombic Zr_6_Ta_2_O_17_ phase without any secondary phase. The zoom-in view of characteristic peak (111) in Fig. 3B shows that the diffraction peak shifts slightly to the left, indicating that the lattice constant of Zr_6_Ta_2_O_17_ ceramics slightly increases with rising temperature [68]. Simultaneously, the gradual splitting of the peaks does not occur in each characteristic peak of Zr_6_Ta_2_O_17_ ceramics. This confirms that temperature would not induce the phase transition of Zr_6_Ta_2_O_17_ ceramics during TPB experiments, including the ferroelastic phase transition [34].

**Fig. 4.**
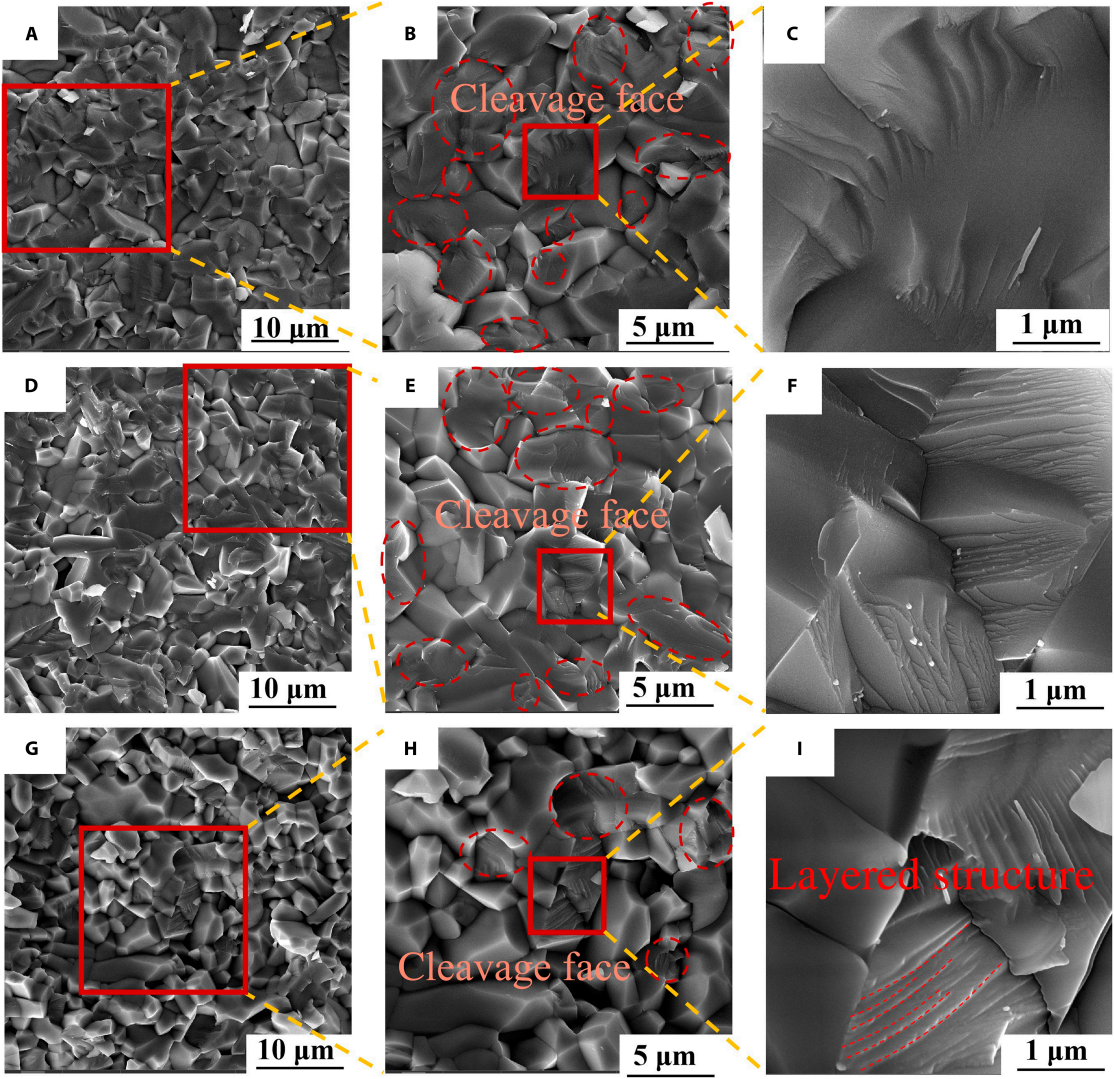
Fracture morphology and magnification after the TPB test at 200°C (A to C), 600°C (D to F), and 1,200°C (G to I).

The fracture morphologies of Zr_6_Ta_2_O_17_ ceramics after in situ TPB tests at 200, 600, and 1,200 °C are shown in Fig. 4. The relatively flat fracture morphology with no dimples suggests that the fracture process of Zr_6_Ta_2_O_17_ ceramics is more susceptible to brittle fracture. This indicates that there is almost no macroscopic plastic deformation. It is consistent with the results of load–deflection curves in Fig. 2A, C, and E. Meanwhile, the enlarged fracture morphology (Fig. 4C, F, and I and Fig. S4) observed after TPB tests at different temperatures is similar to the appearance of cleavage fracture in brittle materials like granite or low carbon martensitic steel [69,70]. Cleavage fracture is a type of brittle fracture that occurs along a specific crystal plane, known as a transgranular fracture [46]. The surface damage morphology of grain also proves that the fracture mode of Zr_6_Ta_2_O_17_ ceramics includes transgranular and intergranular fracture. The mixed fracture mode suggests that the Zr_6_Ta_2_O_17_ ceramics possess exceptional strength and toughness. It is worth noting that the cleavage face of Zr_6_Ta_2_O_17_ ceramics at 1,200°C appears the smallest density and a unique layered structure (Fig. 4H and I). According to Rice–Thomson theory [71], the emission dislocation of ceramic leads to the passivation of crack tip, reducing the possibility of brittle cleavage of cracks, and the density of cleavage face decreases accordingly. Meanwhile, according to Cottrell–Petch theory [72], dislocations tend to produce dislocation pile-up and tensile stress on the cleavage face [73], which is likely to form typical Shockley SFs. When the SF energy of the material is low, it is difficult for dislocations to continue to cross-slip [74], and then the systematic energy increases, thereby significantly improving the strength and stiffness of the material. Therefore, the abnormal phenomenon of the elastic modulus and fracture strength of Zr_6_Ta_2_O_17_ ceramics at high temperature is most likely due to structural changes at the atomic scale, even in cases where there is no obvious difference in the overall fracture mode.

### Unusual TEM phenomenon in entropy-stabilized Zr_6_Ta_2_O_17_ ceramics

**Fig. 5.**
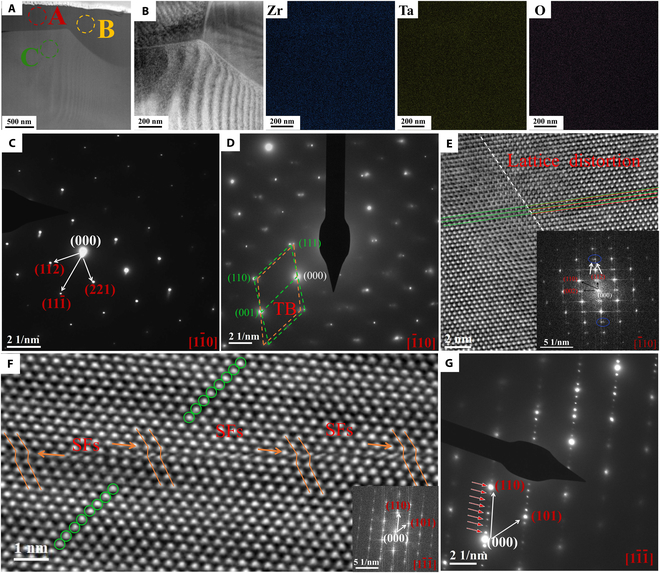
TEM observation of Zr_6_Ta_2_O_17_ ceramics after the TPB test at 200°C. (A) STEM image of TEM specimen. (B) Magnified HAADF-STEM image of (A), and all elements are homogeneously distributed without composition segregation. (C) SAED patterns of region A in (A). (D) SAED patterns of region B in (A). (E) Magnified AC-STEM image of region B in (A), and the inset FFT pattern is indicative of a twin structure. (F) Magnified AC-STEM image of region C in (A) and the inset FFT pattern. (G) SAED patterns of region C in (A).

The atomic-scale microstructure of the fracture surface at different temperatures was investigated using high-resolution transmission electron microscopy (HRTEM) and aberration-corrected scanning transmission electron microscopy (AC-STEM), which are used to reveal the abnormal elastic mechanism of Zr_6_Ta_2_O_17_ ceramics. The TEM observation of Zr_6_Ta_2_O_17_ ceramics after the TPB test at 200 °C is presented in Fig. 5. The high-angle annular dark field (HAADF) image in Fig. 5B exhibits obvious stripe-like structures of different directions around the grain boundary. Scanning transmission electron microscopy–energy-dispersive spectrometer (STEM-EDS) mapping shows that Zr, Ta, and O elements are evenly distributed after the TPB test, without phase transition or element segregation (Fig. 5B). The (112), (221), and $\left(11\bar{1}\right)$ planes of Zr_6_Ta_2_O_17_ (JCPDS 42-0060) under the $\left[1\bar{1}0\right]$ zone axis are clearly observed in the selected-area electron diffraction (SAED) diagram of Fig. 5C, indicating that there is no apparent lattice distortion observed in region A of Zr_6_Ta_2_O_17_ ceramics after the TPB test at 200 °C. Nevertheless, the SAED patterns (green and yellow dashed lines in Fig. 5D) in region B of Fig. 5A show the formation of deformation twins (DTs) under $\left[\bar{1}10\right]$ zone axis. The AC-STEM image in the same region (red and green lines in Fig. 5E) clearly reveals that this area exhibits local distortion attributed to a dislocation, and the inset fast Fourier transform (FFT) image still shows the diffraction characteristics of twins. Combined with the results of in situ HT-XRD and post-test XRD without phase transition, it is confirmed that under the suitable stress conditions, the dislocation movement reverses the stacking sequence of some atom layers and forms a stable twin nucleus [75]. Therefore, the lattice resistance (P-N force) encountered by the movement of partial dislocations [76] requires a higher critical stress for transfer slipping on the twin boundary plane [77], which contributes positively to the mechanical properties of the ceramics. The AC-STEM image (yellow solid line in Fig. 5F) of region C reveals the dislocation pile-up and the formation of SFs, which further promotes the generation of DTs and inhibits the cross-slip of dislocations [78]. Surprisingly, the atomic arrangement exhibits 8-fold periodic ordered atomic structure, as shown in green circle of Fig. 5F. Meanwhile, 8-fold periodic ordered electron diffraction spots on the $\left[1\bar{1}\bar{1}\right]$ zone axis can also be observed in the corresponding SAED (Fig. 5G), which is similar to the long-period structural modulation (LPSO) [79–81] observed in some strengthened alloys or the parent phase structure modified by the ordered superstructure [82]. A series of lattice distortions such as the transition of atomic-scale chemical disorder–ordering and the generation of DTs simultaneously enhance the mechanical properties, including strength and plasticity of the materials [83]. On the other hand, the improvement of chemical order is also a key factor for the material to exhibit the Elinvar-like effect [30].

**Fig. 6.**
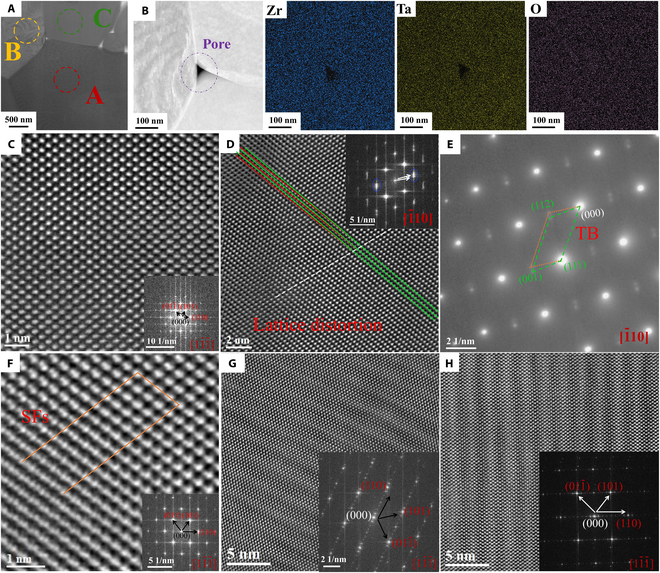
TEM observation of Zr_6_Ta_2_O_17_ ceramics after the TPB test at 600 °C. (A) STEM image of TEM specimen. (B) Magnified HAADF-STEM image of (A), and all elements are homogeneously distributed without composition segregation. (C) Magnified AC-STEM image of region A in (A), and the inset image is FFT pattern. (D) Another magnified AC-STEM image of region A in (A) exhibits local lattice distortion, and the inset image is FFT patterns (the blue circle presents twin nature). (E) SAED patterns of region A close to region B in (A) exhibits DTs. (F) Magnification AC-STEM image of region A in (A), and the inset image is FFT patterns. (G) Medium-magnification AC-STEM image of region B in (A), and the inset image is FFT pattern. (H) Medium-magnification AC-STEM image of region C in (A), and the inset image is FFT patterns.

The TEM images of Zr_6_Ta_2_O_17_ ceramics after the TPB test at 600°C are presented in Fig. 6. The HAADF image and EDS mapping results exhibit distinct grain boundaries surrounding the pores with no element segregation (Fig. 6B). The AC-STEM images of different areas of region A in Fig. 6A are shown in Fig. 6C to F. The overall arrangement of the atoms in Fig. 6C is relatively regular, and the FFT pattern (in the lower right corner) demonstrates that there is no obvious lattice distortion. However, the lattice structure in Fig. 6D is clearly distorted under the $\left[\bar{1}10\right]$ zone axis, similar to the phenomenon observed in Fig. 5D. The FFT (inset in Fig. 6D) and SAED patterns (green and yellow dashed lines in Fig. 6E) of this area reveal the diffraction characteristics of DTs. Excitingly, the formation of SFs (yellow solid line in Fig. 6F) is also noticeable in AC-STEM image particularly in region A near to region B, and the degree of lattice distortion is further enhanced. It is noteworthy that the splitting of the atomic column is a significant factor contributing to the formation of SFs in this region [84]. The AC-STEM image in region B (Fig. 6G) and the FFT pattern obtained under the same $\left[1\bar{1}\bar{1}\right]$ zone axis (inset in Fig. 6G) confirm that this region exhibits a periodic ordered trend. The AC-STEM image and corresponding FFT pattern (Fig. 6H) of region C in Fig. 6A indicate that the lattice in this region is also twisted into a periodic ordered structure. The further enhancement of the degree of order structure optimizes mechanical properties [83]. On the one hand, it compensates for the negative effect of the thermal expansion coefficient of the ceramic on the elastic modulus, thereby ensuring that the material still has the Elinvar-like effect; on the other hand, the combined influence of enhanced chemical order and lattice distortion, such as SFs, may not compromise other mechanical properties, such as fracture strength.

**Fig. 7.**
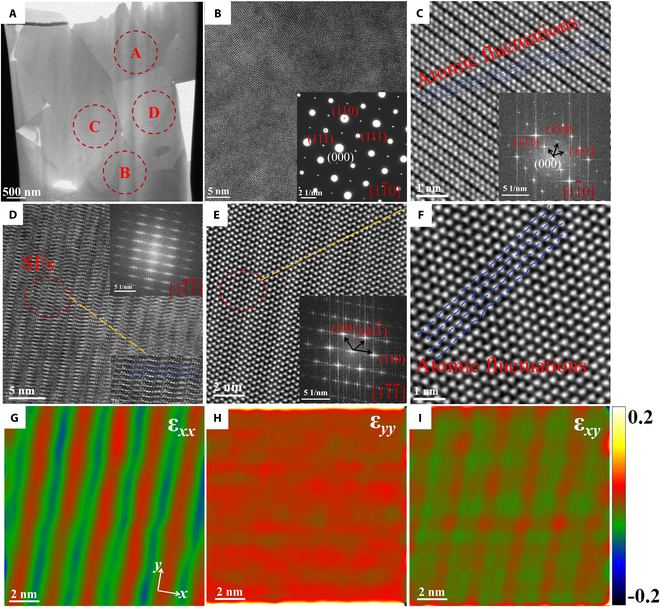
TEM observation of Zr_6_Ta_2_O_17_ ceramics after the TPB test at 1,200°C. (A) STEM image of TEM specimen. (B) Magnified HRTEM image of region A in (A), and inset SAED pattern belongs to region A in (A). (C) Magnified AC-STEM image of region A in (A), and the inset image is FFT pattern. (D) Magnified HRTEM image of region C in (A), the lower right inset image is magnified patterns of SFs, and the upper right inset image is FFT patterns. (E) AC-STEM image of region C in (A), and the inset image is the FFT pattern. (F) Magnified AC-STEM image in (E). (G to I) GPA images of strain distributions *ε_xx_*, *ε_yy_*, and *ε_xy_* of (E).

The TEM images of Zr_6_Ta_2_O_17_ ceramics after the TPB test at 1,200°C are presented in Fig. 7. The results of HAADF-STEM and STEM-EDS show no element segregation after the TPB test at 1,200°C (Fig. S8B). This indicates that Zr_6_Ta_2_O_17_ ceramics maintain excellent stability at high temperature. The HRTEM images in region A are shown in Fig. 7B. In addition to 2 obvious crystal planes of (110) and (111), extra diffraction spots are observed in the SAED patterns along the $\left[1\bar{1}0\right]$ zone axis (inset in Fig. 7B, lower right corner). Meanwhile, the superlattice electron diffraction pattern with periodic order along the zone axis also indicates the improvement in the degree of crystal order, revealing that the disordered crystal lattice of Zr_6_Ta_2_O_17_ ceramics is significantly distorted to an obvious ordered structure under the combined effects of temperature, stress, and Zr–Ta element substitution [85] compared with the TEM images of untested specimen at room temperature (Fig. S5). Different from the observations at 200 and 600°C, the AC-STEM image (Fig. 7C) shows a unique nanostriped superstructure along the (111) crystal plane of $\left[1\bar{1}0\right]$ zone axis in region A. It should be noted that SFs can also be observed in the enlarged HRTEM and AC-STEM images (Fig. 7D and E and Fig. S8C to G) of the 3 regions B, C, and D in Fig. 7A. The light and dark stripe structure (Fig. S8D) and the bright and dark spots in the FFT pattern (the inset image in Fig. S8D) in region B display a trend where the lattice continues to distort, forming a multi-period ordered structure. Interestingly, the magnified AC-STEM images exhibit that the atoms undergo an unprecedented ordered fluctuations [86–89] (Fig. 7C and F and Fig. S8E). Compared with the geometric phase analysis (GPA) internal stress maps of the 25°C specimen (Fig. S7I to L), the strain maps of region C (Fig. 7E) show that the atomic strain fields distribute uniformly in vertical (*y*) direction, but locally concentrated in orderly fluctuating atoms in horizontal (*x*) and shear direction, where alternate tensile and compressive atomic strain fields can be found (Fig. 7G to I). It is speculated that SFs formed by the orderly fluctuating atoms in different directions of atoms may intercept and form Lomer Cottrell Locks or Hirth Locks at the SFs interceptions [90–92] in the Zr_6_Ta_2_O_17_ structure after the TPB test at 1,200°C, and the difficulty of dislocation cross-slipping is further increased. The ordered SFs in this ordered structure further improve the crystal order degree, optimizing the plasticity, strength, and other mechanical properties of Zr_6_Ta_2_O_17_ ceramics. The substantial increase in the degree of atomic-scale order has also led to a more obvious Elinvar-like effect, as well as a sharp rise in the elastic modulus [30]. All in all, the ordered transitions or atomic fluctuations are present in all grains of the Zr_6_Ta_2_O_17_ specimens, but only in those that underwent the TPB test at 1,200°C. More importantly, the TEM observations of Zr_6_Ta_2_O_17_ ceramics after 1,200°C without the TPB test (Fig. S6) and after the TPB test at 25, 200, and 600°C (Fig. S7) provide clearer evidence that mechanical deformation is the primary factor, leading to the lattice distortion of Zr_6_Ta_2_O_17_ ceramics. The temperature can further enhance the degree of lattice distortion and atomic-scale chemical ordering of Zr_6_Ta_2_O_17_ ceramics. In theory, strong lattice distortion or chemical ordering at the atomic scale would locally disturb the Peierls potential, increase the lattice friction stress [93], and dominate the dislocation slip barrier. Furthermore, the relative thickness of different regions (Fig. S9) proves the uniformity of focused ion beam (FIB) specimens thickness, which can exclude the influence of relative thickness inhomogeneity on atomic structure. Therefore, the Elinvar-like effect of high-temperature mechanical properties of Zr_6_Ta_2_O_17_ ceramics is attributed to the highly distorted lattice structure and varied atomic-scale chemical order under the simultaneous action of stress and temperature [30].

The HAADF-STEM images of the fracture surface after TPB tests at different temperatures proved that the material had no phase transformation or precipitation. Figures 5 and 6 and Figs. S5 to S7 show that mechanical deformation dominates the lattice distortion of dislocations in the local region of Zr_6_Ta_2_O_17_ material, which induces the generation of stable twin nucleus and SFs, accompanied by the transition of atomic-scale chemical disorder to ordering. Surprisingly, the STEM results at 600°C show in particular the significant contribution of the splitting of atomic columns in the generation of SFs. As the temperature further increases to 1,200°C, mechanical deformation and high temperature jointly induce strong lattice distortion or chemical ordering at the atomic scale in all regions. Furthermore, ordered SFs and unique atomic ordered fluctuations further enhance the degree of order of the system, resulting in a strong Elinvar-like effect with a significantly abnormal increase in elastic modulus. Therefore, the Elinvar-like effect of the high-temperature mechanical properties of Zr_6_Ta_2_O_17_ ceramics is caused by lattice distortion (DTs, SFs) and the disorder–order transition of chemical order at the atomic scale under the simultaneous action of stress and temperature.

## Conclusion

In conclusion, the Zr_6_Ta_2_O_17_ ceramic material with excellent comprehensive mechanical properties is selected based on the first-principles results, and then we present an unconventional microstructural feature observed in entropy-stabilized Zr_6_Ta_2_O_17_ ceramics via in situ TPB tests from 25 to 1,200°C. The elastic modulus of Zr_6_Ta_2_O_17_ ceramics remains relatively constant within the temperature range of 25 to 600°C but exhibits an abnormal increase at higher temperatures. The load–deflection curves and scanning electron microscopy (SEM) images reveal that brittle fracture predominates as the fracture mode in Zr_6_Ta_2_O_17_ ceramics. The TEM observations confirm the highly distorted lattice structure and disorder–order transition of chemical ordering in Zr_6_Ta_2_O_17_ ceramics, primarily influenced by mechanical deformation with a supplementary effect of temperature.

The unique structural characteristics of Zr_6_Ta_2_O_17_ ceramics contribute to their remarkable energy barrier against dislocation movements, showcasing exceptional resistance to deformation and exhibiting the Elinvar-like effect. Additionally, the structural disorder is further reduced due to the occurrence of chemical ordered transformation and atomic fluctuations in all grains during the high-temperature section, leading to further increase in elastic modulus and an enhanced Elinvar effect. Furthermore, the special crystal structure contributes to an improvement in mechanical properties, including plasticity and strength, suggesting that Zr_6_Ta_2_O_17_ ceramics may maintain outstanding service performance at high temperatures. To the best of our knowledge, these properties are unparalleled in comparison to traditional ceramics and even reported alloys. This unique high-temperature strengthening ceramics hold potential applications in high-performance protective materials, especially for new TBCs operating at elevated higher temperature (>1,400 °C) in high-thrust aero-engine.

## Materials and Methods

### Specimen preparation

The Zr_6_Ta_2_O_17_ ceramics were synthesized through a high-temperature solid-state reaction, utilizing ZrO_2_ (purity ≥ 99.99%, China New Metal Materials Technology Co. Ltd.) and Ta_2_O_5_ (purity ≥ 99.99%, China New Metal Materials Technology Co. Ltd.) as raw materials. The powders with a chemical composition of 85.71 mol % ZrO_2_ and 14.29 mol % Ta_2_O_5_ were precisely weighed and subsequently mixed with ethanol through planetary ball milling for 20 h. The resulting slurry was dried at 80°C for 12 h and then reacted at 1,600°C for 20 h to produce pre-sintered Zr_6_Ta_2_O_17_ powder. Following this, the Zr_6_Ta_2_O_17_ powder was ground and sieved using a 500-mesh sieve to obtain the final powders for pressing. Uniaxial cold pressing at 200 MPa was employed to prepare strip specimens with dimensions of 40 mm × 6 mm × 2.5 mm. The as-pressed specimens were subjected to a drying process in an oven at 60°C for 24 h to alleviate internal stress generated during pressing. Finally, the specimens underwent pressureless sintering in air at 1,600 °C for 20 h, followed by furnace cooling, resulting in dense Zr_6_Ta_2_O_17_ ceramics with a density of 97%.

### High-temperature in situ TPB characterization

The elastic modulus and fracture strength of Zr_6_Ta_2_O_17_ ceramics were determined using a high-temperature TPB method at various temperatures (25 to 1,200°C). The surface displacement and strain evolution of the ceramics during TPB tests were monitored in situ using a DIC system. A high-temperature speckle pattern was applied to the specimen for obtaining displacement and strain distributions during the TPB tests, following the procedures outlined in our previous work [46]. A CCD camera with a resolution of 1,624 × 1,236 pixels from GOM Co., Germany, was positioned in front of the specimen’s cross-section to record the deformation process. The sampling frequency of the CCD camera was set at one image per second. For the TPB experiments, the Zr_6_Ta_2_O_17_ specimen was secured in the fixture with a clamp span of 27 mm. The specimens were isothermally preserved for 10 min before initiating the TPB experiment to ensure uniform temperature distribution. The loading rate during the TPB test was set at 0.03 mm/min. At least 3 Zr_6_Ta_2_O_17_ specimens were tested for each temperature. The elastic modulus (*E*) and fracture strength (*σ*_b_) were calculated using the established methods [46,65]

$$E=\frac{{\mathrm{KL}}^{3}}{4{\mathrm{bh}}^{3}}$$ (5)

$$\sigma_{b}=\frac{3P_{b}L}{2{\mathrm{bw}}^{2}}$$ (6)

where *K* is the slope of the tangent to the initial linear part of load–deflection curve, *L* is the span of the clamp, *b* is the width of the specimen, and *h* is the thickness of the specimen. *P*_b_ is the critical load corresponding to the specimen fracture in the TPB tests.

### Microstructure and compositional characterization

The phase structure of Zr_6_Ta_2_O_17_ ceramics was detected using XRD with CuKα radiation, employing a scanning range of 10° to 90° and a scanning rate of 4°/min. The phase structures of Zr_6_Ta_2_O_17_ ceramics at different temperatures were analyzed by in situ HT-XRD (SmartLab SE). Microstructural observations were performed by SEM (TESCAN MIRA3 LMH) equipped with EDS (Oxford X MAX20) for elemental analysis. The TEM specimen was nanoprocessed by FIB (FEI Scio 2 HiVac). The cross-section sheet was thinned to a thickness of 80 nm with an accelerating voltage of 30 kV and a maximum current of 2.5 nA, followed by fine polishing under the conditions of 5-kV accelerating voltage and a small current of 16 pA. It is worth noting that all specimens were not processed using Ar ion milling to prevent structural damage. The microstructure of the fracture section was analyzed using HRTEM (FEI Talos F200X G2, Hillsboro, USA), with HAADF images obtained at a height of 88 mm and an acceptance angle of 50 to 200 mrad. The specimens were characterized by HAADF-STEM using a super-x spectrometer. The STEM-EDS images were acquired with a pixel count exceeding 30 million pixels for each image to ensure image quality. Additionally, the atomic-scale structure of the Zr_6_Ta_2_O_17_ fracture section was finely characterized using double spherical AC-STEM (Titan Themis G2 60-300, Thermo-Fisher, MA, USA), with HAADF images acquired at an acceptance angle of 48 to 200 mrad. GPA was conducted using Gatan digital micrographs software [94].

### Simulation details

The mechanical properties of solid-state physics were investigated by first-principles calculation. The VASP software, based on density functional theory (DFT), was employed to study the crystal structure and elastic properties [95,96] of A_6_B_2_O_17_ compounds. The electron-ion exchange was represented by the projection augmented wave (PAW) description [97], and the exchange-correlation functional was constructed by using the generalized gradient approximation (GGA) proposed by Perdew–Burke–Ernzerhof (PBE) [98]. The cutoff energy value for expanding the electron wave function in the plane-wave basis set was taken as 550 eV for A_6_B_2_O_17_ compounds. The reciprocal-space integration over the Brillouin zone was done with 1 × 8 × 8 Monkhorst–Pack *k*-point grid [61]. The convergence criteria were set to ensure a total convergence energy of less than 1.0 meV/atom, a residual stress of less than 0.02 eV/Å, and a maximum stress deviation of less than 0.02 GPa. The conjugate gradient method was selected to optimize the atomic position, the shape, and volume of the crystal unit cell. In order to obtain more accurate energy, the final self-consistent static state of the crystal unit cell was calculated by the tetrahedral smear method with Blcohl correction [99]. In this study, the crystal structure of orthorhombic A_6_B_2_O_17_ compounds was initially referenced, followed by Zr substitution at the A site and Ta substitution at the B site. Ultimately, the cell model of A_6_B_2_O_17_ (A = Zr, Ta, B = Hf, Nb) compounds was obtained.

## Data Availability

Data that support the findings presented in this manuscript can be provided upon reasonable request by contacting the corresponding author.
