# Supplementary material for: Elinvar-Like Effect Induced by High Lattice Distortion in Zr6Ta2O17 Ceramics
Source: Research (Wash D C). 2024 Aug 5;7:0436. doi: 10.34133/research.0436 (PMC12327365; doi:10.34133/research.0436)
Supplement: Supplementary 1 — Figs. S1 to S8 Table S1 [file research.0436.f1.docx]

Supplementary Information

**Elinvar-like effect induced by** **high lattice distortion in Zr_6_Ta_2_O_17_ ceramics**

Xiaopeng Hu ^a, b †^, Qing Liu ^a, b †^, Sai Liu ^a, b^, Yu Zou ^a, b^, Jinwei Guo ^a, b^, Junyao Wu ^a, b^, Wang Zhu ^a, b *^, Zengsheng Ma ^a, b *^

*^a^ Key Laboratory of Key Film Materials & Application for Equipment (Hunan province), School of Materials Science and Engineering, Xiangtan University, Xiangtan, Hunan 411105, China*

*^b^ Key Laboratory of Low Dimensional Materials and Application Technology of Ministry of Education, School of Materials Science and Engineering, Xiangtan University, Xiangtan, Hunan 411105, China*

**Section 1: Microstructure and element distributions of Zr_6_Ta_2_O_17_ ceramics**

**Section 2: Load-deflection curves at different temperatures**

**Section 3: Phase stability after TPB test at different temperatures**

**Section 4: Fracture morphology after TPB tests at different temperatures**

**Section 5: TEM observation of the original Zr_6_Ta_2_O_17_ ceramics before TPB test**

**Section 6: TEM observation of Zr_6_Ta_2_O_17_ ceramics after 1200°C without TPB test**

**Section 7: TEM observation of Zr_6_Ta_2_O_17_ ceramics after TPB test at 25°C**

**Section 8: TEM observation on other region of Zr_6_Ta_2_O_17_ ceramics after TPB test at 1200°C**

**Section 9: Investigation on the relative thickness of different regions at different temperatures**

**Section 1: Microstructure and element distributions of Zr_6_Ta_2_O_17_ ceramics**


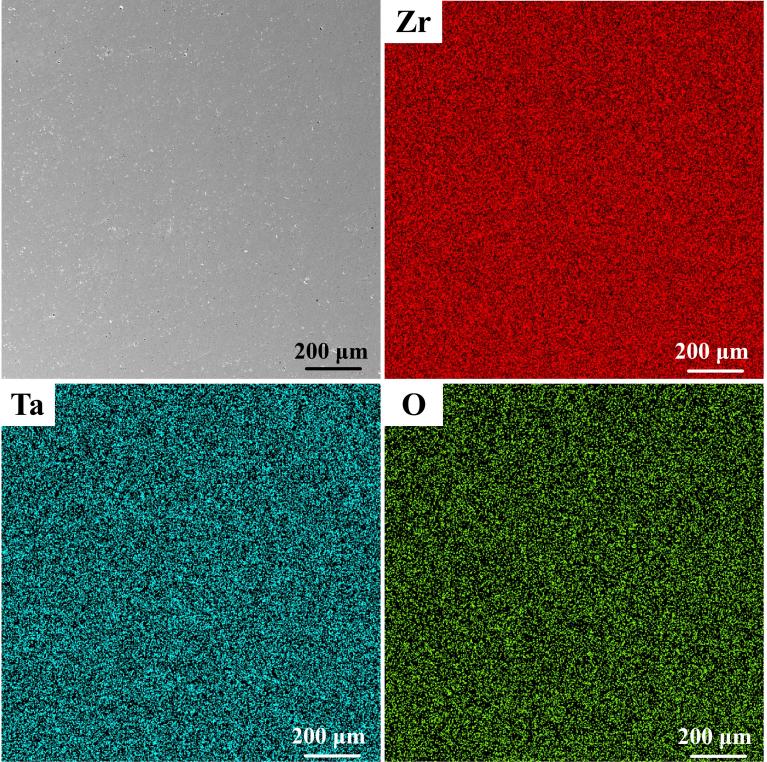


Fig. S1│Microstructure and element distributions. Scanning electron microscope images of polished surface of Zr_6_Ta_2_O_17_ ceramics before TPB test.

The surface microstructure of Zr_6_Ta_2_O_17_ ceramics is shown in Fig. S1. It can be seen that the Zr_6_Ta_2_O_17_ ceramic is dense and the element distribution is uniform. The bulk density and porosity of the Zr_6_Ta_2_O_17_ ceramics were measured by Archimedes principle with distilled water as the impregnation solution. In order to avoid the discreteness of the data, three specimens were tested. The bulk density of Zr_6_Ta_2_O_17_ ceramics is 7.19 g/cm^3^, and the relative density is 98%.

**Section 2: Load-deflection curves at different temperatures.**


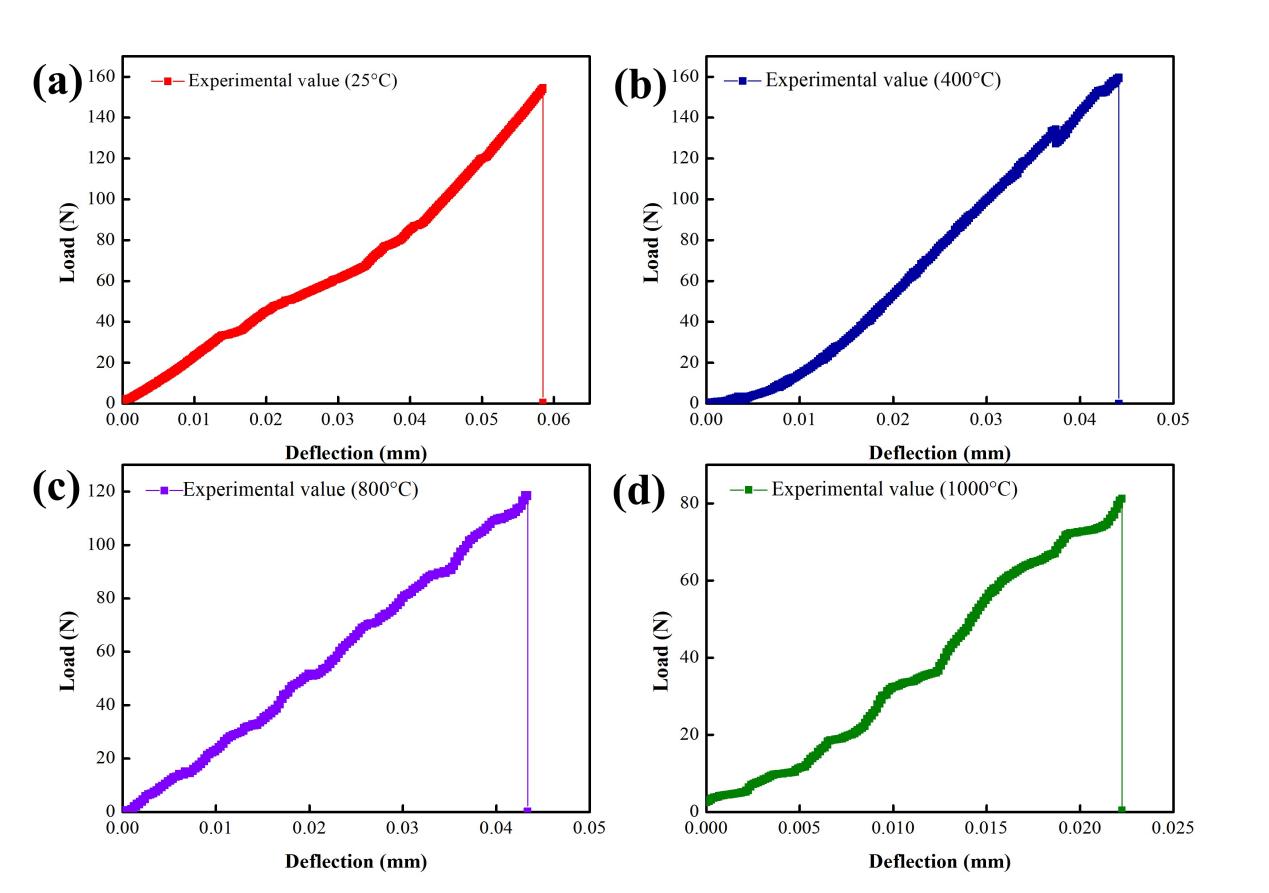


Fig. S2│ The load-deflection curves of Zr_6_Ta_2_O_17_ ceramics acquired by the TPB tests at different temperatures: (a) 25°C; (b) 400°C; (c) 800°C and (d) 1000°C.

Table S1 The elastic modulus and fracture strength of the Zr_6_Ta_2_O_17_ ceramics at different temperatures.

| Temperature (°C) | Elastic modulus (GPa) | | Fracture strength (GPa) | |
| --- | --- | --- | --- | --- |
|  | mean value | standard error | mean value | standard error |
| 25 | 170.384 | 8.477 | 203.365 | 4.167 |
| 200 | 184.525 | 4.211 | 215.928 | 2.124 |
| 400 | 161.333 | 6.230 | 157.098 | 3.066 |
| 600 | 141.812 | 9.411 | 111.592 | 4.628 |
| 800 | 164.384 | 4.787 | 116.187 | 2.185 |
| 1000 | 211.409 | 2.261 | 149.273 | 1.349 |
| 1200 | 278.796 | 6.195 | 165.202 | 3.008 |

The load-deflection curves Zr_6_Ta_2_O_17_ ceramics at 25°C, 400°C, 800°C and 1000°C are shown in Fig. S2. The elastic modulus and fracture strength calculated by the slope of the load-deflection curves and the critical fracture load are shown in Table S1. The elastic modulus value conforms to the Elinvar effect below 800°C, and shows an abnormal increase trend at high temperature, the maximum elastic modulus is up to 1.6 times that of room temperature.

**Section 3: Phase stability after TPB test at different temperatures**

Fig. S3│ The XRD patterns of Zr_6_Ta_2_O_17_ ceramics after TPB tests at different temperatures.

The XRD patterns of Zr_6_Ta_2_O_17_ ceramics after TPB test at different temperatures are shown in Fig. S3. Zr_6_Ta_2_O_17_ ceramics still maintain high temperature stability after high temperature TPB test, and there is no obvious splitting and movement of the peak position.

**Section 4: Fracture morphology after TPB tests at different temperatures**


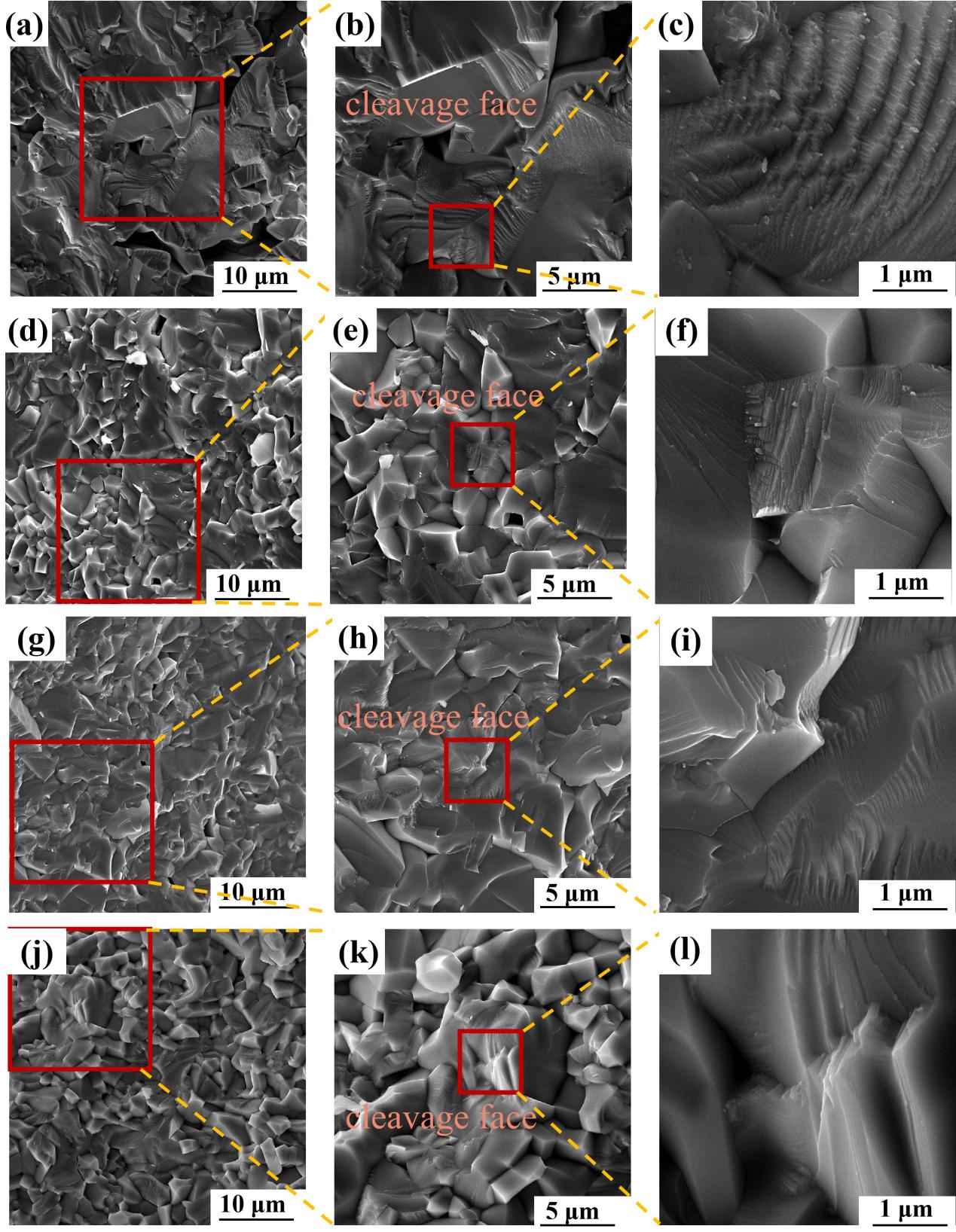


Fig. S4│ The fracture morphology of Zr_6_Ta_2_O_17_ ceramics after TPB tests at different temperatures: (a-c) 25°C; (d-f) 400°C; (g-i) 800°C; (d-f) 1000°C.

The fracture morphology of Zr_6_Ta_2_O_17_ ceramics after TPB tests at different temperatures is shown in Fig. S4. The fracture mode is a synergistic effect of transgranular and intergranular fractures. No obvious dimples appear in the fracture surface, and obvious cleavage steps appear. In a broad sense, It still exhibits brittle fracture behavior. However, according to the larger size image, the frequency of cleavage surface decreases obviously with the increase in temperature.

**Section 5: TEM observation of the original Zr_6_Ta_2_O_17_ ceramics before TPB test**


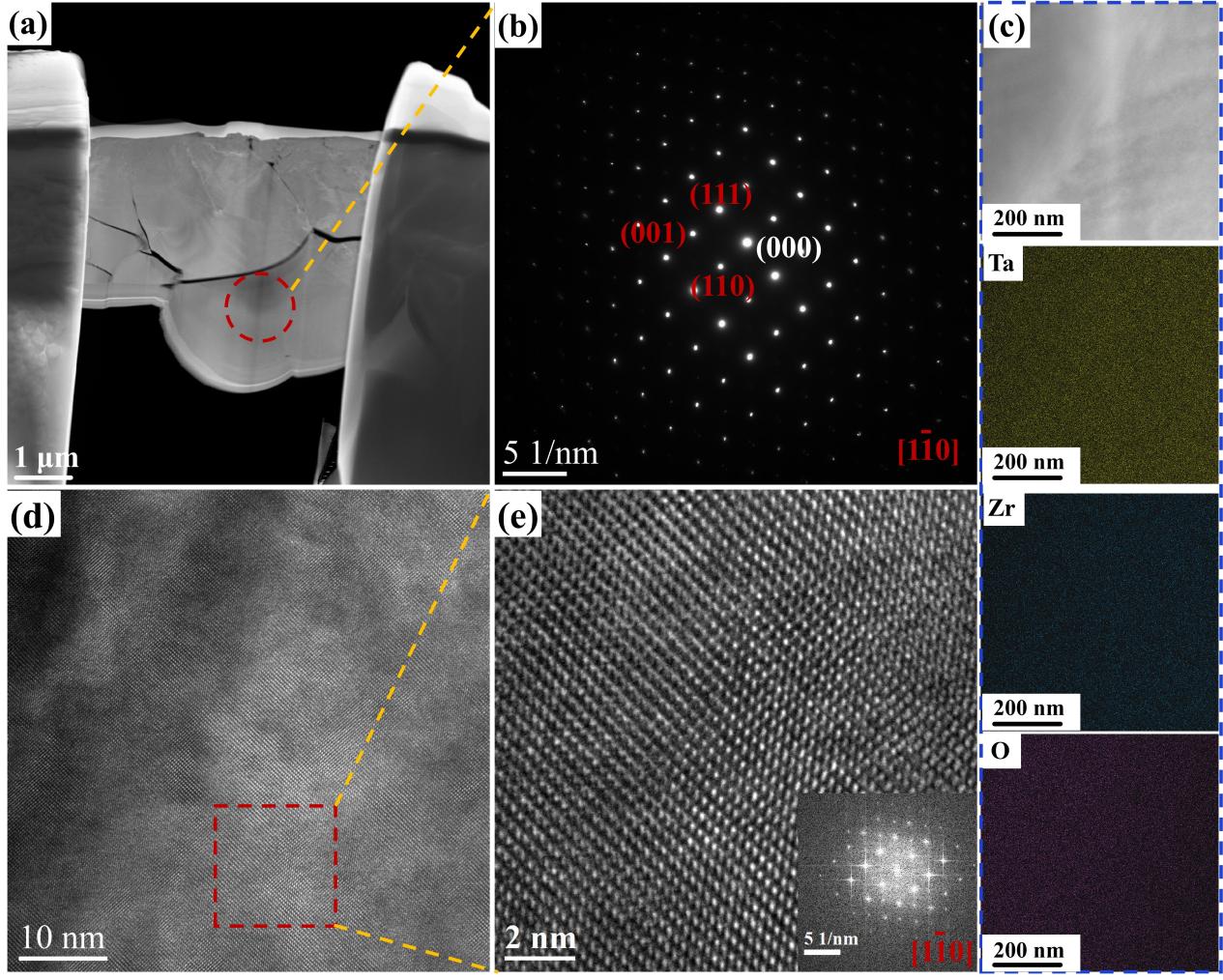


Fig. S5│TEM observation of Zr_6_Ta_2_O_17_ ceramics before TPB test at room temperature: (a) HAADF-STEM image of TEM specimen; (b) SAED patterns of the red circle region in Fig. S5(a); (c) magnified HAADF-STEM images of the red circle region in Fig. S5(a), and all elements are homogeneously distributed without composition segregation; (d) magnified HRTEM image of the red circle region in Fig. S5(a); (e) magnified image of the red square in Fig. S5(d), and the inset image is FFT patterns of Fig. S5(e).

Zr_6_Ta_2_O_17_ ceramic before TPB test at room temperature was investigated by TEM observation, as shown in Fig. S5. The magnified HRTEM image (Fig. S5(e)) reveals that the atoms are arranged neatly without obvious lattice distortion, and the FFT patterns (insets in Fig. 5(e)) exhibited single-crystal nature of a single crystal grain, which is similar to the literature report [1, 2]. HAADF-STEM and STEM-EDS images confirmed that the elements of ceramics have good uniformity.

**Section 6: TEM observation of Zr_6_Ta_2_O_17_ ceramic after 1200°C without TPB test**


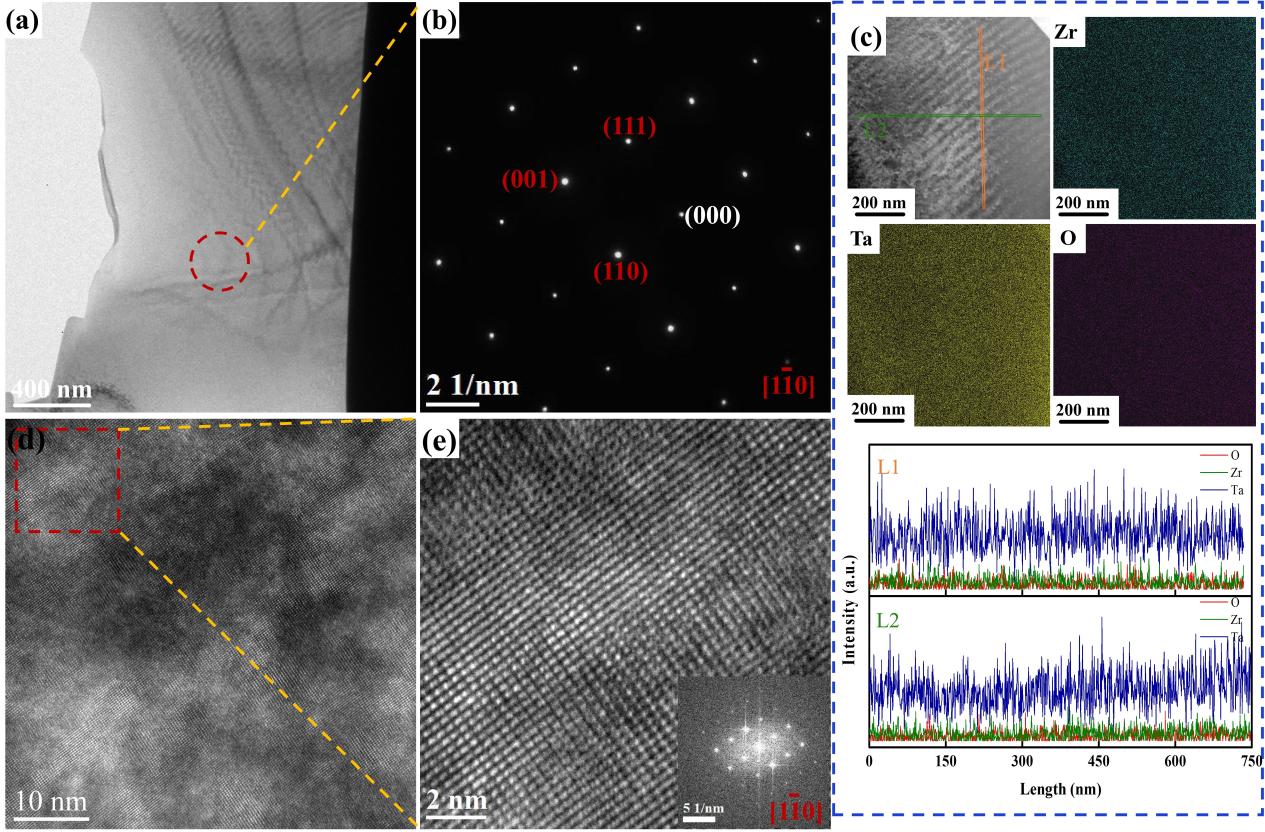


Fig. S6│TEM observation of Zr_6_Ta_2_O_17_ ceramics after 1200°C without TPB test: (a) HAADF-STEM image of TEM specimen; (b) SAED patterns of the red circle region in Fig. S6(a); (c) magnified HAADF-STEM images of the red circle region in Fig. S6(a), and all elements along L_1_ and L_2_ are homogeneously distributed without composition segregation; (d) magnified HRTEM image of the red circle region in Fig. S6(a); (e) magnified image of the red square in Fig. S6(d), and the inset image is FFT patterns of Fig. S6(e).

Zr_6_Ta_2_O_17_ ceramic after 1200°C without TPB test was investigated by TEM observation, as shown in Fig. S6. Compared to Fig. S5, the lattice has not been obviously distorted, and SEAD pattern also shows that there are no deformation twins and stacking faults, which still presents single-crystal nature of a single crystal grain. HAADF images, STEM-EDS images and line scan images show that there is still no segregation, enrichment and phase transformation, indicating that Zr_6_Ta_2_O_17_ ceramic maintains good high-temperature stability after being subjected to high temperatures. This proves that temperature is not the main factor affecting the lattice distortion of Zr_6_Ta_2_O_17_ ceramic.

**Section 7:** **TEM observation of Zr_6_Ta_2_O_17_ ceramics after TPB test at 25°C**


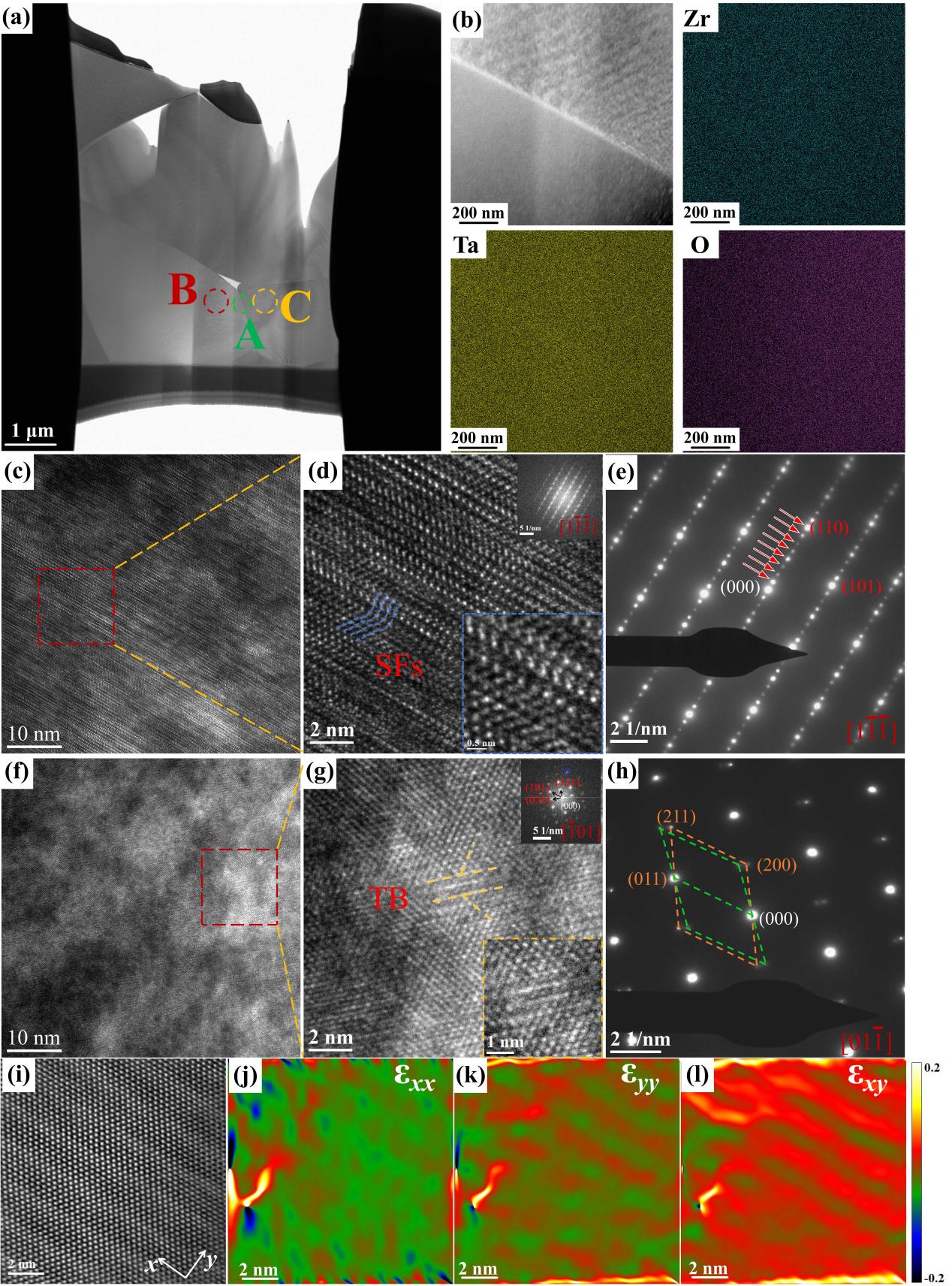


Fig. S7│TEM observation of Zr_6_Ta_2_O_17_ ceramics after TPB test at 25°C: (a) HAADF-STEM image of TEM specimen; (b) magnified HAADF-STEM images of region A in Fig. S7(a), and all elements in the STEM-EDS images are homogeneously distributed without composition segregation; (c) magnified HRTEM image of region B in Fig. S7(a); (d) magnified image of the red square in Fig. S7(c), the lower right inset image is magnified patterns of SFs in Fig. S7(d), and the upper right inset image is FFT patterns of Fig. S7(d); (e) SAED patterns of region B in Fig. S7(a); (f) magnified HRTEM image of region C in Fig. S7(a); (g) magnified image of the red square in Fig. S7(f), the lower right inset image is magnified patterns of TB in Fig. S7(g), the upper right inset image is FFT patterns of Fig. S7(g); (h) SAED patterns of region C in Fig. S7(a); (i) AC-STEM image of region B in Fig. S7(a); (j-l) GPA images of strain distributions *ε_xx_, ε_yy_* and *ε_xy_* of Fig. S7(i).

In order to explore the effect of mechanical action on lattice distortion, TEM observation of Zr_6_Ta_2_O_17_ ceramics after TPB test at 25°C is shown in Fig. S7. Although the HAADF and STEM-EDS images prove that there is still no segregation or phase transition, the HRTEM and the AC-STEM images in Figs. S7(c, i) show obvious nano-stripe structure, indicating that the lattice distortion may gradually change into chemical order structure. Although the HRTEM in Fig. S7(f) is similar to that of Fig. S5 and Fig. S6, the microstructure magnification image (Fig. S7(g)) indicates the occurrence of deformation twins. More interestingly, the stacking faults can be observed in Fig. S7(d). The existence of stacking faults and deformation twins can be also proved by SAED patterns in Fig. S7(e, h). It is worth noting that the geometric phase analysis (GPA) strain maps (Figs. S7(j-l)) of Fig. S7(i) illustrate that the atomic structure in the region c exhibits significant internal stress due to lattice distortion. Combined with Fig. S6, the effect of temperature can be further excluded, proving that mechanical deformation is the dominant factor of atomic distortion.

**Section 8: TEM observation on other region of Zr_6_Ta_2_O_17_ ceramics after TPB test at 1200°C**


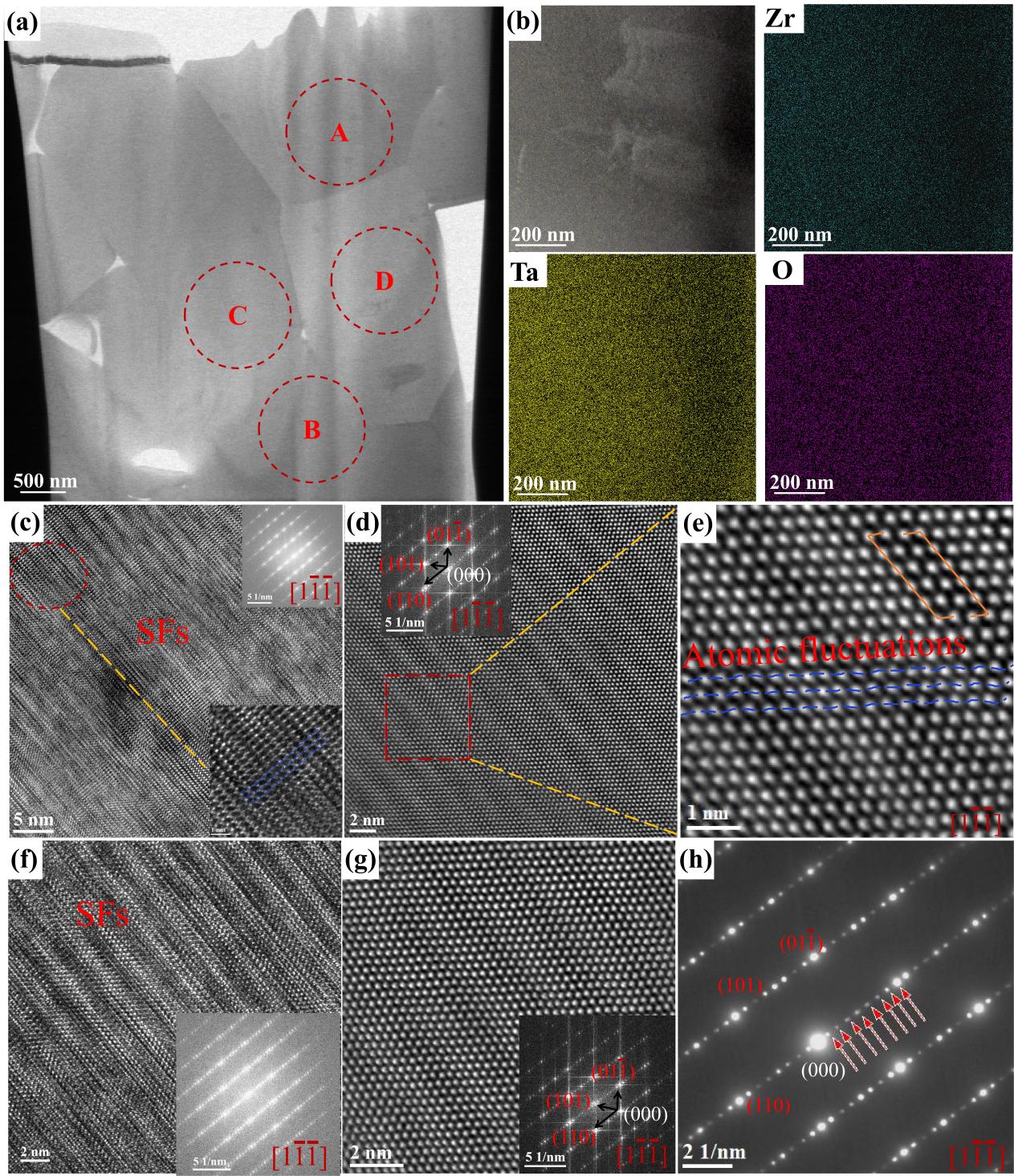


Fig. S8│TEM observation of Zr_6_Ta_2_O_17_ ceramics after TPB test at 1200°C: (a) HAADF-STEM image of TEM specimen; (b) magnified HAADF-STEM image of the region B in Fig. S8(a), and all elements are homogeneously distributed without composition segregation; (c) magnified HRTEM image of the region B in Fig. S8(a), the lower right inset image is magnified pattern of SFs in Fig. S8(c), and the upper right inset image is FFT pattern of Fig. S8(c); (d) magnified AC-STEM image of the region B in Fig. S8(a), and the inset image is FFT pattern; (e) magnified AC-STEM image of Fig. S8(d); (f) magnified HRTEM image of the region D in Fig. S8(a), and the inset image is FFT patterns; (g) AC-STEM image of the region D in Fig. S8(a), and the inset image is the FFT pattern; (h) SAED patterns of the region D in Fig. S8(a).

The TEM images of Zr_6_Ta_2_O_17_ ceramics after TPB test at 1200°C are shown in Fig. S8. The results of HAADF-STEM and STEM-EDS present no element segregation after TPB test at 1200°C in Figs. S8(b), indicating Zr_6_Ta_2_O_17_ ceramics maintain excellent phase stability after high temperature TPB test. The HRTEM (Fig. S8(b), AC-STEM (Fig. S8(c)) images and the corresponding FFT in the B region show the emergence of ordered structures and SFs. At the meantime, the ordered fluctuations of atoms can be still observed in Fig S8(e). In addition, similar SFs and ordered structures (Figs. S8(c-e)) were observed in the D region, indicating that the degree of atomic distortion and ordering is further improved in the Zr_6_Ta_2_O_17_ ceramics after TPB test at 1200°C compared with the TEM results of 25°C, 200°C and 600°C.

**Section 9:** **Investigation on the relative thickness of different regions at different temperatures.**


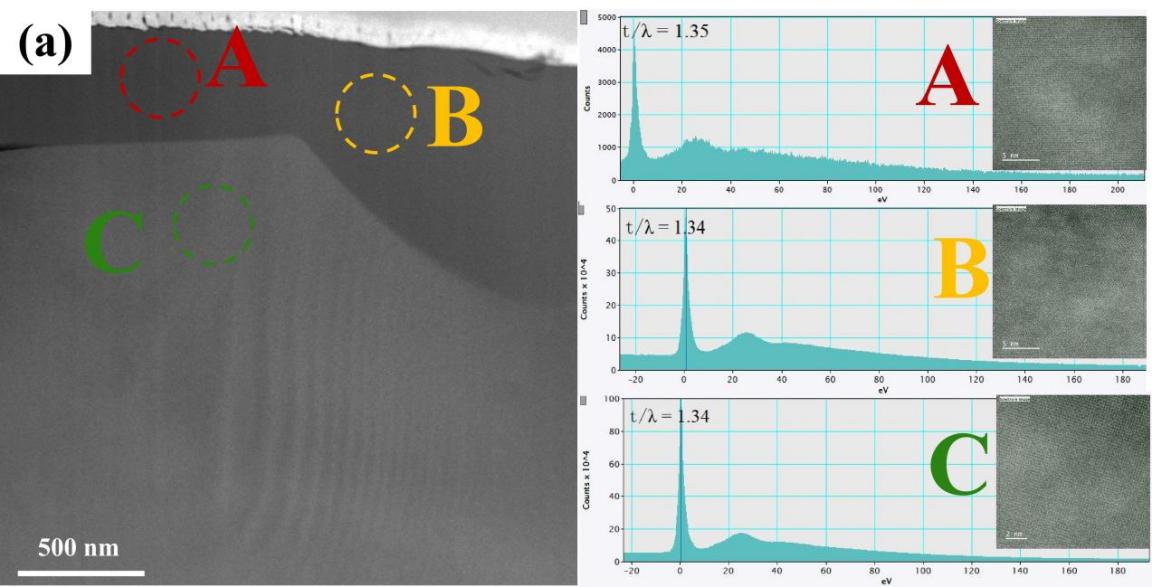


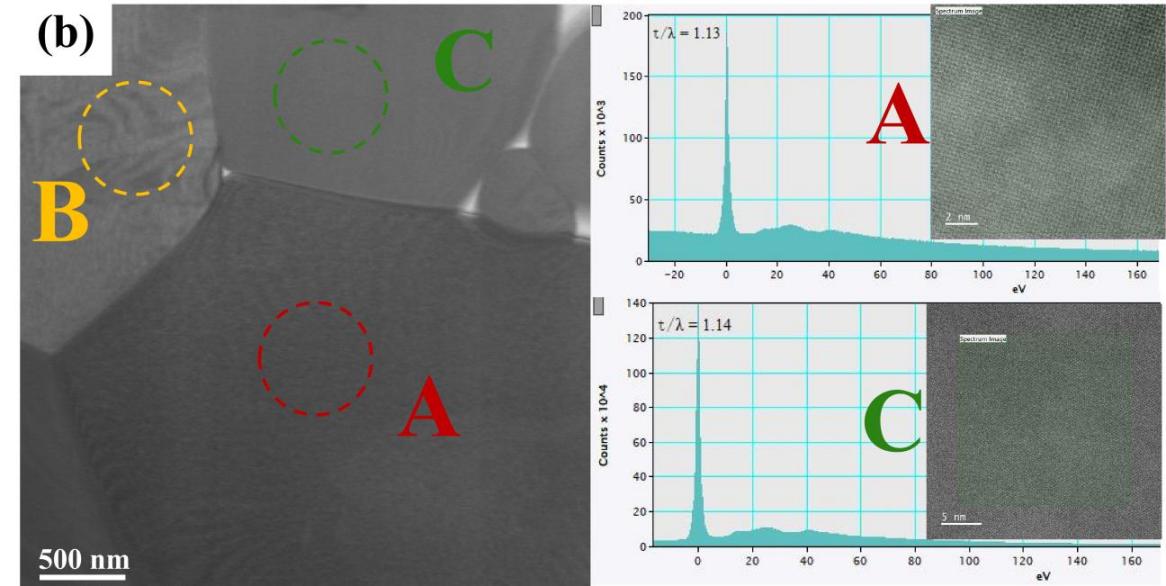

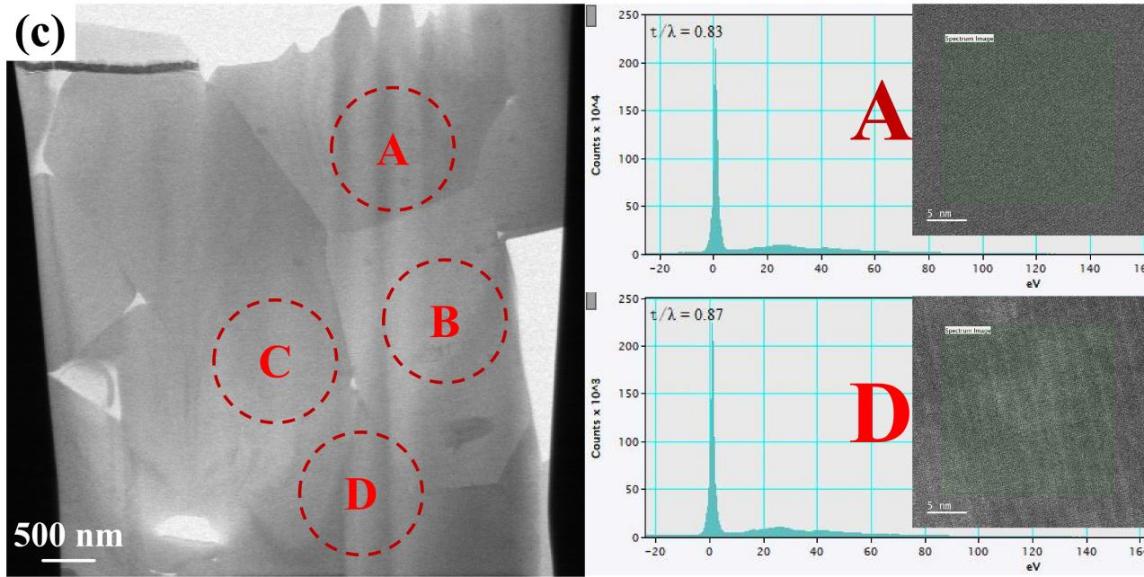


Fig. S9│Relative thickness of different positions at different temperatures: the t / λ map acquired by EELS of Zr_6_Ta_2_O_17_ ceramics after the TPB tests at (a) 200°C, (b) 600°C, (c) 1200°C, respectively.

As shown in Fig. S9, the Log-ratio acquired by EELS was used to explore the relative thickness of the FIB specimens after the TPB tests at different temperatures. The t/λ values for the A, B and C regions of the 200°C FIB specimens were 1.35, 1.34, and 1.34, respectively. The overall thickness of the FIB specimens is relatively uniform, and the t/λ values in different regions remain stable after undergoing TPB tests at 600°C and 1200°C. The reason for the larger contrast can be attributed to lattice distortion, and it can be excluded that the influence of thickness is the cause of the contrast.

**References**

[1] Q. Liu, X.P. Hu, W. Zhu, J.W. Guo, Z.Y. Tan, Effects of Ta_2_O_5_ content on mechanical properties and high-temperature performance of Zr_6_Ta_2_O_17_ thermal barrier coatings, J. Am. Ceram. Soc., 104 (2021) 6533-6544.

[2] J. Zhang, S. Wang, W. Li, J. Jiang, Synthesis and characterization of nanocrystalline Hf_6_Ta_2_O_17_ ternary oxides by solvothermal method, Mater Res Express., 6 (2019): 125069.
